# Some limit theorems for weighted negative quadrant dependent random variables with infinite mean

**DOI:** 10.1186/s13660-018-1655-5

**Published:** 2018-03-20

**Authors:** Fuqiang Ma, Jianmin Li, Tiantian Hou

**Affiliations:** College of Mathematics and Statistics, Pingdingshan University, Pingdingshan, China

**Keywords:** 60F05, 60F15, Strong law, Weak law of large numbers, Pairwise negative quadrant dependent sequence

## Abstract

In the present paper, we will investigate weak laws of large numbers for weighted pairwise NQD random variables with infinite mean. The almost sure upper and lower bounds for a particular normalized weighted sum of pairwise NQD nonnegative random variables are established also.

## Introduction

With Markov’s truncation method, Kolmogorov got a weak law of large numbers for independent identically random variables with a necessary and sufficient condition, which is called the Kolmogorov–Feller weak law of large numbers.

### Theorem 1.1

([1])

*Let* $\{X_{i}, i\ge 1\}$ *be a sequence of i*.*i*.*d*. *random variables with partial sums* $S_{n}=X_{1}+\cdots +X_{n}$. *Then*

$$ \frac{S_{n}-n\mathbb {E}X_{1}1\{\vert X_{1} \vert \le n\}}{n} \xrightarrow{\mathbb {P}} 0,\quad \textit{as } n\to \infty, $$

*if and only if*

1.1 $$ x\mathbb {P}\bigl(\vert X_{1} \vert >x\bigr)\to 0, \quad \textit{as } x\to \infty . $$

The theorem states the condition of the mean’s existence is not necessary, and St. Petersburg game (see [2]) and Feller game (see [3]), which are well known as the typical examples, are formulated by a nonnegative random variable *X* with the tail probability

1.2 $$ \mathbb {P}(X > x)\asymp x^{-\alpha } $$

for each fixed $0 <\alpha \le 1$, where $a_{n}\asymp b_{n}$ denotes

$$ 0< \liminf_{n\to \infty }\frac{a_{n}}{b_{n}}\le \limsup _{n\to \infty }\frac{a _{n}}{b_{n}}< \infty . $$

Nakata [4] considered truncated random variables and studied strong laws of large numbers and central limit theorems in this situation. In [5], Nakata studied the weak laws of large numbers for weighted independent random variables with the tail probability (1.2) and explored the case that the decay order of the tail probability is −1. In this paper, we shall consider the more general random sequence with infinite mean.

Let us recall the concept of negative quadrant dependent (NQD) random variables, which was introduced by Lehmann [6].

### Definition 1.1

Two random variables *X* and *Y* are said to be NQD if for any $x,y\in \mathbb {R}$

$$ \mathbb {P}(X\le x, Y\le y)\le \mathbb {P}(X\le x)\mathbb {P}(Y\le y). $$

A sequence of random variables $\{X_{n}, n\ge 1\}$ is said to be pairwise NQD if, for all $i,j\in N$, $i\ne j$, $X_{i}$ and $X_{j}$ are NQD.

Because pairwise NQD includes the independent, NA (negatively associated), NOD (negatively orthant dependent) and LNQD (linearly negative quadrant dependent), it is a more general dependence structure. It is necessary to study its probabilistic properties.

### Definition 1.2

A finite sequence $\{X_{1}, \ldots , X_{n}\}$ of random variables is said to be NA if for any disjoint subsets *A*, *B* of $\{1, \ldots , n \}$ and any real coordinatewise nondecreasing functions *f* on $\mathbb {R}^{\vert A \vert }$ and *g* on $\mathbb {R}^{\vert B \vert }$,

1.3 $$ \operatorname{Cov}\bigl(f(X_{k}, k\in A), g(X_{k}, k\in B) \bigr) \le 0 $$

whenever the covariance exists, where $\vert A \vert $ denotes the cardinality of *A*. An infinite family of random variables is NA if every finite subfamily is NA.

The concept of a NA sequence was introduced by Joag-Dev and Proschan [7], and it is easy to see that a sequence of NA random variables is a pairwise NQD sequence.

In the present paper, we suppose that all random variables satisfy the condition

1.4 $$ \mathbb {P}\bigl(\vert X \vert >x\bigr)\asymp x^{-\alpha } \quad \text{for a fixed } 0< \alpha \le 1. $$

In Sect. 2, we will investigate weak laws of large numbers for weighted pairwise NQD random variables with the common distribution (1.4). The almost sure upper and lower bounds for a particular normalized weighted sum of pairwise NQD nonnegative random variables will be established in Sect. 3. Throughout this paper, the symbol *C* represents positive constants whose values may change from one place to another.

## Weak law of large numbers

In this section, we extend the corresponding results in Nakata [5] from the case of i.i.d. random variables to pairwise NQD random variables.

### Main results

We state our weak law of large numbers for different weighted sums of pairwise NQD random variables.

#### Theorem 2.1

*Let* $\{X_{i}, i\ge 1\}$ *be a sequence of pairwise NQD random variables whose distributions satisfy*

$$ \mathbb {P}\bigl(\vert X_{j} \vert >x\bigr)\asymp x^{-\alpha } \quad \textit{for } j\ge 1 $$

*and*

$$ \limsup_{x\to \infty } \sup_{j\ge 1}x^{\alpha }\mathbb {P}\bigl(\vert X_{j} \vert >x\bigr)< \infty . $$

*If there exist two positive sequences* $\{a_{j}\}$ *and* $\{b_{j}\}$ *satisfying*

2.1 $$ \sum_{j=1}^{n} a_{j}^{\alpha }=o\bigl(b_{n}^{\alpha }\bigr), $$

*then it follows that*

2.2 $$ \lim_{n\to \infty }\frac{1}{b_{n}}\sum _{j=1}^{n} a_{j} \biggl( X_{j}- \mathbb {E}X _{j}1 \biggl\{ \vert X_{j} \vert \le \frac{b_{n}}{a_{j}} \biggr\} \biggr) =0 \quad \textit{in probability}. $$

*In particular*, *if there exists a constant* *A* *such that*

$$ \lim_{n\to \infty }\frac{1}{b_{n}}\sum_{j=1}^{n} a_{j}\mathbb {E}X_{j}1 \biggl\{ \vert X_{j} \vert \le \frac{b_{n}}{a_{j}} \biggr\} =A, $$

*then we have*

$$ \lim_{n\to \infty }\frac{1}{b_{n}}\sum_{j=1}^{n} a_{j} X_{j}=A \quad \textit{in probability}. $$

From Theorem 2.1 and by using the same methods as in Nakata [5] to calculate the constant *A*, we can obtain the following four corollaries for the pairwise NQD random variables.

#### Corollary 2.1

*Under the assumptions of Theorem* 2.1, *if* $0<\alpha <1$, *then we have*

$$ \lim_{n\to \infty }\frac{1}{b_{n}}\sum_{j=1}^{n} a_{j} X_{j}=0 \quad \textit{in probability}. $$

#### Corollary 2.2

*Let* $\{X_{i}, i\ge 1\}$ *be a sequence of nonnegative pairwise NQD random variables whose distributions satisfy*

$$ \mathbb {P}\bigl(\vert X_{j} \vert >x\bigr)= (x+q_{j})^{-1} \quad \textit{for } x\ge 0. $$

*If* $q_{j}\ge 1$ *for any positive integer* *j*,*and*

2.3 $$ Q_{n}:=\sum_{j=1}^{n}q_{j}^{-1} \to \infty , \quad \textit{as } n\to \infty . $$

*Then we have*

$$ \lim_{n\to \infty }\frac{\sum_{j=1}^{n} q_{j}^{-1}X_{j}}{Q_{n}\log Q _{n}}=1 \quad \textit{in probability}. $$

#### Corollary 2.3

*Let us suppose the assumptions of Corollary* 2.2 *and* $q_{j}=j$ *in Eq*. (2.3). *Then*, *for any* $\gamma >-1$ *and real* *δ*, *we have*

$$ \lim_{n\to \infty }\frac{\sum_{j=1}^{n} j^{-1}(\log j)^{\gamma }( \log \log j)^{\delta }X_{j}}{(\log n)^{\gamma +1}(\log \log n)^{ \delta +1}}=\frac{1}{\gamma +1} \quad \textit{in probability}. $$

#### Corollary 2.4

*Let* $\{X_{i}, i\ge 1\}$ *be a sequence of pairwise NQD random variables whose common distribution satisfies* (1.4) *with* $\alpha =1$. *If there exists a real* *Q* *such that*

$$ \lim_{x\to \infty }\frac{\mathbb {E}X1(\vert X \vert \le x)}{\log x}=Q. $$

*Then*, *for each real* $\beta >-1$ *and slowly varying sequence* $l(n)$, *it follows that*

$$ \lim_{n\to \infty }\frac{\sum_{j=1}^{n} j^{\beta }l(j)X_{j}}{n^{ \beta +1}l(n)\log n}=\frac{Q}{1+\beta } \quad \textit{in probability}. $$

#### Theorem 2.2

*Let* $\{X_{i}, i\ge 1\}$ *be a sequence of pairwise NQD random variables whose distributions satisfy*

$$ \mathbb {P}\bigl(\vert X_{j} \vert >x\bigr)\asymp x^{-\alpha } \quad \textit{for } j\ge 1 $$

*and*

$$ \limsup_{x\to \infty } \sup_{j\ge 1}x^{\alpha }\mathbb {P}\bigl(\vert X_{j} \vert >x\bigr)< \infty . $$

*If there exist two positive sequences* $\{a_{j}\}$ *and* $\{b_{j}\}$ *satisfying*

2.4 $$ \bigl( \log^{2} n \bigr) \sum _{j=1}^{n} a_{j}^{\alpha }=o \bigl(b_{n}^{\alpha }\bigr). $$

*Then it follows that*

2.5 $$ \lim_{n\to \infty }\frac{1}{b_{n}}\max _{1\le k\le n}\Biggl\vert \sum_{j=1} ^{k} a_{j} \biggl( X_{j}-\mathbb {E}X_{j}1 \biggl\{ \vert X_{j} \vert \le \frac{b_{n}}{a_{j}} \biggr\} \biggr) \Biggr\vert =0 \quad \textit{in probability}. $$

#### Corollary 2.5

*Under the assumptions of Theorem* 2.2, *if* $0<\alpha <1$, *then we have*

$$ \lim_{n\to \infty }\frac{1}{b_{n}}\max_{1\le k\le n}\Biggl\vert \sum_{j=1} ^{k} a_{j} X_{j}\Biggr\vert =0 \quad \textit{in probability}. $$

#### Remark 2.1

If $\{X_{i}, i\ge 1\}$ is a sequence of NA random variables satisfying the assumptions of Theorem 2.2, then from the maximal inequality of NA random variables (see [8, Theorem 2]), the condition (2.4) can be weakened by (2.1).

### Proofs of Theorem 2.1 and Theorem 2.2

We first give some useful lemmas.

#### Lemma 2.1

([5])

*If a random variable* *X* *satisfies* (1.4), *then it follows that*

$$ \mathbb {E}\bigl(\vert X \vert 1\bigl(\vert X \vert \le x\bigr)\bigr)\asymp \textstyle\begin{cases} x^{1-\alpha },& \textit{if } 0< \alpha < 1, \\ \log x,& \textit{if } \alpha =1, \end{cases} $$

*and*

$$ \mathbb {E}\bigl(\vert X \vert ^{2}1\bigl(\vert X \vert \le x\bigr) \bigr)\asymp x^{2-\alpha } \quad \textit{for } 0< \alpha \le 1. $$

#### Lemma 2.2

([6])

*Let* $\{X_{n},n\ge 1\}$ *be a sequence of pairwise NQD random variables*. *Let* $\{f_{n}, n\ge 1\}$ *be a sequence of increasing functions*. *Then* $\{f_{n}(X_{n}), n\ge 1\}$ *is a sequence of pairwise NQD random variables*.

#### Lemma 2.3

([9])

*Let* $\{X_{n},n\ge 1\}$ *be a sequence of pairwise NQD random variables with mean zero and* $\mathbb {E}X_{n}^{2}<\infty $, *and* $T_{j}(k)= \sum_{i=j+1}^{j+k} X_{i}$, $j\ge 0$. *Then*

$$ \mathbb {E}\bigl(T_{j}(k)\bigr)^{2}\le C\sum _{i=j+1}^{j+k} \mathbb {E}X_{i}^{2}, \qquad \mathbb {E}\max_{1\le k\le n} \bigl(T_{j}(k)\bigr)^{2}\le C\log^{2} n\sum_{i=j+1}^{j+n} \mathbb {E}X _{i}^{2}. $$

#### Proof of Theorem 2.1

For any $1\le i\le n$, let us define

$$ Y_{ni}=-b_{n}1(a_{i}X_{i}< -b_{n})+a_{i}X_{i}1 \bigl(a_{i}\vert X_{i} \vert \le b_{n}\bigr)+b _{n}1(a_{i}X_{i}>b_{n}) $$

and

$$ Z_{ni}=(a_{i}X_{i}+b_{n})1(a_{i}X_{i}< -b_{n})+(a_{i}X_{i}-b_{n})1(a _{i}X_{i}>b_{n}). $$

Then from Lemma 2.2 it follows that $\{Y_{ni}, 1\le i\le n, n \ge 1\}$ and $\{Z_{ni}, 1\le i\le n, n\ge 1\}$ are both pairwise NQD, and

$$ \sum_{i=1}^{n} a_{i} X_{i}=\sum_{i=1}^{n} (Y_{ni}+Z_{ni}). $$

Furthermore, let us define $X_{ni}= X_{i}1(a_{i}\vert X_{i} \vert \le b_{n})$, then the limit (2.2) holds if we show

2.6 $$\begin{aligned}& \lim_{n\to \infty } \frac{1}{b_{n}}\sum _{i=1}^{n} (Y_{ni}-\mathbb {E}Y_{ni})=0 \quad \text{in probability}, \end{aligned}$$

2.7 $$\begin{aligned}& \lim_{n\to \infty } \frac{1}{b_{n}}\sum _{i=1}^{n} (a_{i}X_{i}- Y_{ni})=0 \quad \text{in probability}, \end{aligned}$$

and

2.8 $$ \lim_{n\to \infty } \frac{1}{b_{n}}\sum _{i=1}^{n} (a_{i}\mathbb {E}X_{ni}- \mathbb {E}Y _{ni})=0. $$

Using the proof of Lemma 2.2 in [4], we get

$$ \frac{1}{b^{2}_{n}}\sum_{i=1}^{n} a_{i}^{2}\mathbb {E}\bigl[ X_{i}^{2}1\bigl(a _{i}\vert X_{i} \vert \le b_{n}\bigr) \bigr] \to 0 $$

and

$$ \sum_{i=1}^{n} \mathbb {P}\bigl(a_{i} \vert X_{i} \vert \ge b_{n}\bigr)\to 0. $$

From Lemma 2.1 and Lemma 2.3, we have

$$\begin{aligned} \frac{1}{b^{2}_{n}}\operatorname{Var} \Biggl( \sum_{i=1}^{n}(Y_{ni}-\mathbb {E}Y_{ni}) \Biggr) &\le \frac{C}{b^{2}_{n}}\sum _{i=1}^{n}\mathbb {E}Y_{ni}^{2} \\ & \le \frac{C}{b ^{2}_{n}}\sum_{i=1}^{n} a_{i}^{2}\mathbb {E}\bigl[ X_{i}^{2}1 \bigl(a_{i}\vert X_{i} \vert \le b_{n}\bigr) \bigr] \\ &\quad {}+C\sum_{i=1}^{n} \mathbb {P}\bigl(a_{i}\vert X_{i} \vert \ge b_{n}\bigr) \to 0, \end{aligned}$$

which implies (2.6). Similarly, for any $r>0$, we have

$$\begin{aligned} \mathbb {P}\Biggl( \frac{1}{b_{n}}\Biggl\vert \sum_{i=1}^{n} Z_{ni}\Biggr\vert >r \Biggr)&\le \mathbb {P}\Biggl( \bigcup_{i=1}^{n} \bigl\{ a_{i}\vert X_{i} \vert >b_{n}\bigr\} \Biggr) \\ &\le \sum_{i=1}^{n} \mathbb {P}\bigl( a_{i}\vert X_{i} \vert >b_{n} \bigr) \to 0, \end{aligned}$$

which yields (2.7). Finally, (2.8) holds since

$$ \frac{1}{b_{n}}\Biggl\vert \sum_{i=1}^{n} (a_{i}\mathbb {E}X_{ni}- \mathbb {E}Y_{ni})\Biggr\vert \le C \sum_{i=1}^{n}\mathbb {P}\bigl(a_{i} \vert X_{i} \vert >b_{n}\bigr)\to 0. $$

Based on the above discussions, the desired results are obtained. □

#### Proof of Theorem 2.2

By a proof similar to that of Theorem 2.1, it is enough to show

2.9 $$\begin{aligned}& \lim_{n\to \infty } \frac{1}{b_{n}}\max _{1\le k\le n}\Biggl\vert \sum_{i=1} ^{k} (Y_{ni}-\mathbb {E}Y_{ni})\Biggr\vert =0 \quad \text{in probability}, \end{aligned}$$

2.10 $$\begin{aligned}& \lim_{n\to \infty } \frac{1}{b_{n}}\max _{1\le k\le n}\Biggl\vert \sum_{i=1} ^{k} (a_{i}X_{i}- Y_{ni})\Biggr\vert =0 \quad \text{in probability}, \end{aligned}$$

and

2.11 $$ \lim_{n\to \infty } \frac{1}{b_{n}}\max _{1\le k\le n}\Biggl\vert \sum_{i=1} ^{k} (a_{i}\mathbb {E}X_{ni}- \mathbb {E}Y_{ni}) \Biggr\vert =0. $$

From Lemma 2.1 and Lemma 2.3, we have

$$\begin{aligned} &\frac{1}{b^{2}_{n}}\mathbb {E}\Biggl( \max_{1\le k\le n}\Biggl\vert \sum_{i=1}^{k} (Y _{ni}-\mathbb {E}Y_{ni})\Biggr\vert ^{2} \Biggr) \\ &\quad \le \frac{C\log^{2} n}{b^{2}_{n}} \sum_{i=1}^{n}\mathbb {E}Y_{ni}^{2} \\ &\quad \le \frac{C\log^{2} n}{b^{2}_{n}} \sum_{i=1}^{n} a_{i}^{2}\mathbb {E}\bigl[ X_{i}^{2}1 \bigl(a_{i}\vert X_{i} \vert \le b_{n}\bigr) \bigr] +C \log^{2} n\sum_{i=1}^{n} \mathbb {P}\bigl(a_{i}\vert X_{i} \vert \ge b_{n} \bigr) \\ &\quad \le \frac{C \log^{2} n}{b^{2}_{n}}\sum_{i=1}^{n} a_{i}^{2}(b_{n}/a_{i})^{2-\alpha }+ \frac{C\log^{2} n}{b_{n}^{\alpha }}\sum_{i=1}^{n} a_{i}^{\alpha } \to 0, \end{aligned}$$

which implies (2.9). Similarly, for any $r>0$, we have

$$ \begin{aligned}\mathbb {P}\Biggl( \frac{1}{b_{n}}\max_{1\le k\le n}\Biggl\vert \sum_{i=1}^{k} Z_{ni}\Biggr\vert >r \Biggr) \le \mathbb {P}\Biggl( \bigcup_{i=1}^{n} \bigl\{ a_{i}\vert X_{i} \vert >b_{n}\bigr\} \Biggr) \le \sum_{i=1}^{n} \mathbb {P}\bigl( a_{i}\vert X_{i} \vert >b_{n} \bigr) \to 0,\end{aligned}$$

which yields (2.10). At last

$$ \frac{1}{b_{n}}\max_{1\le k\le n}\Biggl\vert \sum _{i=1}^{k} (a_{i}\mathbb {E}X_{ni}- \mathbb {E}Y_{ni})\Biggr\vert \le C\sum_{i=1}^{n} \mathbb {P}\bigl(a_{i}\vert X_{i} \vert >b_{n}\bigr) \to 0. $$

Based on the above discussions, the desired result is obtained. □

## One side strong law

Adler [10] considered the almost sure upper and lower bounds for a particular normalized weighted sum of independent nonnegative random variables (see Corollary 2.2). In this section, we extend his work from the independent case to pairwise NQD nonnegative random variables.

### Main results

#### Theorem 3.1

*Let* $\{X_{i}, i\ge 1\}$ *be a sequence of nonnegative pairwise NQD random variables whose distributions satisfy*

$$ \mathbb {P}\bigl(\vert X_{j} \vert >x\bigr)= (x+q_{j})^{-1} \quad \textit{for } x\ge 0 $$

*where* $q_{j}\ge 1$ *for any positive integer* *j*, *and*

3.1 $$ Q_{n}:=\sum_{j=1}^{n}q_{j}^{-1} \to \infty , \quad \textit{as } n\to \infty . $$

*If*

3.2 $$ \sum_{n=1}^{\infty } \frac{q_{n}^{-1}}{Q_{n}\log Q_{n}}=\infty , $$

*then we have*

$$ \limsup_{n\to \infty }\frac{\sum_{j=1}^{n} q_{j}^{-1}X_{j}}{Q_{n} \log Q_{n}}=\infty\quad\textit{almost surely}. $$

#### Theorem 3.2

*Let* $\{X_{i}, i\ge 1\}$ *be a sequence of nonnegative pairwise NQD random variables whose distributions satisfy*

$$ \mathbb {P}(X_{j}>x)= (x+q_{j})^{-1} \quad \textit{for } x \ge 0 $$

*where* $q_{j}\ge 1$ *for any positive integer* *j*, *and*

3.3 $$ Q_{n}:=\sum_{j=1}^{n}q_{j}^{-1} \to \infty , \quad \textit{as } n\to \infty . $$

*If there is a sequence* $\{d_{n}, n\ge 1\}$ *such that*

3.4 $$ \lim_{n\to \infty } \frac{1}{Q_{n}\log Q_{n}}\sum _{j=1}^{n} q^{-1} _{j}\log \bigl(q_{j}^{-1}d_{j}\bigr)=1 $$

*and*

3.5 $$ \sum_{n=1}^{\infty } \frac{q_{n}^{-2}d_{n}\log^{2}n}{Q_{n}^{2}\log^{2}Q _{n}}< \infty , $$

*then we have*

$$ \liminf_{n\to \infty }\frac{1}{Q_{n}\log Q_{n}}\sum _{j=1}^{n} q_{j} ^{-1}X_{j}=1\quad \textit{almost surely.} $$

#### Remark 3.1

For the independent case, the assumption (3.5) can be weakened by the following condition (see [10]):

3.6 $$ \sum_{n=1}^{\infty } \frac{q_{n}^{-2}d_{n}}{Q_{n}^{2}\log^{2}Q_{n}}< \infty . $$

If $\{X_{i}, i\ge 1\}$ is a sequence of NA random variables, then from the maximal inequality of NA random variables (see [8, Theorem 2]), the condition (3.5) can be weakened by (3.6).

#### Remark 3.2

Let us give an example to show that the conditions (3.4) and (3.5) can be satisfied. If we choose $q_{j}^{-1}=j^{\alpha}$, $d_{j}=j/\log^{2} j$ where $\alpha>-1$, then it is easy to show

$$\begin{aligned}& Q_{n}\sim \frac{n^{\alpha+1}}{\alpha+1},\qquad \ Q_{n}\log Q_{n}\sim n^{\alpha+1}\log n, \\& \sum_{i=1}^{n}q_{i}^{-1}\log q^{-1}_{i}=\alpha\sum_{i=1}^{n}i^{\alpha}\log i\sim\frac{\alpha}{\alpha+1}n^{\alpha+1}\log n, \end{aligned}$$

and

$$ \sum_{i=1}^{n}q_{i}^{-1}\log d_{i}=\sum_{i=1}^{n}i^{\alpha}(\log i-2\log\log i)\sim\frac{1}{\alpha+1}n^{\alpha+1}\log n. $$

Hence we have

$$ \frac{1}{Q_{n}\log Q_{n}}\sum_{j=1}^{n} q^{-1}_{j}\log\bigl(q_{j}^{-1}d_{j}\bigr)\to 1 $$

and

$$ \sum_{n=1}^{\infty}\frac{q_{n}^{-2}d_{n}\log^{2}n}{Q_{n}^{2}\log^{2}Q_{n}}=\sum_{n=1}^{\infty}\frac{n^{2\alpha}n}{n^{2(\alpha+1)}\log^{2} n}< \infty. $$

### Proofs of Theorem 3.1 and Theorem 3.2

Before giving our proofs, we need the following useful lemmas.

#### Lemma 3.1

([11])

*Let* $\{A_{n}, n\ge 1\}$ *be a sequence of events*, *such that* $\sum_{n=1}^{\infty }\mathbb {P}(A_{n})=\infty $. *Then*

$$ \mathbb {P}( A_{n}, i.o. ) \ge \limsup_{n\to \infty } \frac{ ( \sum_{k=1}^{n} \mathbb {P}(A_{k}) ) ^{2}}{\sum_{i,k=1}^{n}\mathbb {P}(A_{i}A_{k})}. $$

#### Lemma 3.2

([9])

*Let* $\{X_{n}, n\ge 1\}$ *be pairwise NQD random sequences*. *If*

$$ \sum_{n=1}^{\infty }\log^{2} n \operatorname{Var}(X_{n})< \infty , $$

*then we have*

$$ \sum_{n=1}^{\infty }(X_{n}-\mathbb {E}X_{n}) \quad \textit{converges almost surely.} $$

#### Proof of Theorem 3.1

For any $M>0$, we have

$$ \begin{aligned}\sum_{n=1}^{\infty }\mathbb {P}\biggl( \frac{ q_{n}^{-1}X_{n}}{Q_{n}\log Q_{n}}>M \biggr) = \sum_{n=1}^{\infty } \mathbb {P}\biggl( X_{n}> \frac{MQ_{n}\log Q_{n}}{q_{n}^{-1}} \biggr) =\infty .\end{aligned}$$

Let us define $A_{n}= \{ X_{n}>\frac{MQ_{n}\log Q_{n}}{q_{n}^{-1}} \} $, then we have

$$ \mathbb {P}(A_{i}A_{k})\le \mathbb {P}(A_{i})\mathbb {P}(A_{k}), $$

where we used the fact that $\{X_{n}, n\ge 1\}$ is a sequence of nonnegative pairwise NQD random variables. Hence from Lemma 3.1, we get

$$ \limsup_{n\to \infty }\frac{ q_{n}^{-1}X_{n}}{Q_{n}\log Q_{n}}=\infty\quad\text{almost surely}, $$

which yields

$$ \limsup_{n\to \infty }\frac{ \sum_{i=1}^{n}q_{i}^{-1}X_{i}}{Q_{n} \log Q_{n}}\ge \limsup _{n\to \infty }\frac{ q_{n}^{-1}X_{n}}{Q_{n} \log Q_{n}}=\infty \quad \text{almost surely}. $$

 □

#### Proof Theorem 3.2

For $n\ge 1$, let us define

$$ Y_{n}=X_{n}1(X_{n}\le d_{n})+d_{n}1(X_{n}> d_{n}) $$

and

$$ Z_{n}=(X_{n}-d_{n})1(X_{n}> d_{n}). $$

Then from Lemma 2.2 it follows that $\{Y_{n}, n\ge 1\}$ and $\{Z_{n}, n\ge 1\}$ are both pairwise NQD, and

3.7 $$\begin{aligned} \sum_{i=1}^{n} q_{i}^{-1} X_{i} =\sum _{i=1}^{n} q_{i}^{-1}(Y_{i}+Z_{i}) = \sum_{i=1}^{n} q_{i}^{-1}(Y_{i}- \mathbb {E}Y_{i})+\sum_{i=1}^{n} q_{i}^{-1}Z _{i}+\sum _{i=1}^{n} q_{i}^{-1}\mathbb {E}Y_{i}. \end{aligned}$$

Since

$$\begin{aligned} \mathbb {E}Y_{n}^{2}&\le C \bigl( \mathbb {E}X_{n}^{2}1(X_{n}\le d_{n})+d_{n}^{2} \mathbb {P}(X _{n}>d_{n}) \bigr) \\ &= C \biggl( \int_{0}^{d_{n}} \frac{x^{2}}{(x+q_{n})^{2}}\,dx+d_{n}^{2} \int_{d_{n}}^{\infty }\frac{1}{(x+q _{n})^{2}}\,dx \biggr) \le C d_{n}, \end{aligned}$$

then from the condition (3.5), we have

$$ \sum_{n=1}^{\infty }\frac{q_{n}^{-2}\log^{2} n\operatorname{Var}(Y_{n})}{Q_{n}^{2} \log^{2} Q_{n}}\le \sum _{n=1}^{\infty }\frac{q_{n}^{-2}\log^{2} nd _{n}}{Q_{n}^{2}\log^{2} Q_{n}}< \infty . $$

Thus by Lemma 3.2, we have

$$ \sum_{n=1}^{\infty }\frac{q_{n}^{-1} (Y_{n}-\mathbb {E}Y_{n})}{Q_{n}\log Q _{n}} \quad \text{converges almost surely}, $$

which, by the Kronecker lemma, implies that

3.8 $$ \lim_{n\to \infty }\sum_{i=1}^{n} \frac{q_{i}^{-1} (Y_{i}-\mathbb {E}Y_{i})}{Q _{n}\log Q_{n}}=0 \quad\text{almost surely}. $$

Furthermore, since

$$\begin{aligned} \mathbb {E}Y_{n}&= \mathbb {E}X_{n}1(X_{n}\le d_{n})+d_{n}\mathbb {P}(X_{n}> d_{n}) \\ &= \int_{0}^{d_{n}}\frac{x}{(x+q_{n})^{2}}\,dx+d_{n} \int^{\infty }_{d_{n}}\frac{1}{(x+q _{n})^{2}}\,dx \\ &= \int_{q_{n}}^{d_{n}+q_{n}}\frac{1}{x}\,dx-q_{n} \int_{q_{n}}^{d_{n}+q_{n}}\frac{1}{x^{2}}\,dx+d_{n} \int^{\infty }_{d _{n}}\frac{1}{(x+q_{n})^{2}}\,dx \\ &= \log (1+d_{n}/q_{n})-1+\frac{q _{n}}{d_{n}+q_{n}}+ \frac{d_{n}}{d_{n}+q_{n}} \\ &=\log (1+d_{n}/q_{n}) \end{aligned}$$

then by the condition (3.4) we have

3.9 $$ \lim_{n\to \infty }\sum_{i=1}^{n} \frac{q_{i}^{-1} \mathbb {E}Y_{i}}{Q_{n} \log Q_{n}}=1 \quad\text{almost surely}. $$

Hence, from (3.7), (3.8) and (3.9), we have

3.10 $$ \liminf_{n\to \infty }\sum_{i=1}^{n} \frac{q_{i}^{-1} X_{i}}{Q_{n} \log Q_{n}}\ge \lim_{n\to \infty }\sum _{i=1}^{n} \frac{q_{i}^{-1}(Y _{i}-\mathbb {E}Y_{i})}{Q_{n}\log Q_{n}}+\lim _{n\to \infty }\sum_{i=1}^{n} \frac{q _{i}^{-1}\mathbb {E}Y_{i}}{Q_{n}\log Q_{n}}=1. $$

By Corollary 2.2, we have

3.11 $$ \liminf_{n\to \infty }\sum_{i=1}^{n} \frac{q_{i}^{-1} X_{i}}{Q_{n} \log Q_{n}}\leq 1 \quad\text{almost surely}. $$

So, from (3.10) and (3.11), we have

$$ \liminf_{n\to \infty }\sum_{i=1}^{n} \frac{q_{i}^{-1} X_{i}}{Q_{n} \log Q_{n}}=1\quad\text{almost surely}. $$

 □
